# Clinical Significance of MTHFR C677T and A1298C Polymorphisms in Adult Patients with ALL and NHL

**DOI:** 10.3390/jcm15051796

**Published:** 2026-02-27

**Authors:** Hatice Demet Kiper Unal, Tugba Cetintepe, Roya Gasimli, Alev Garip Acar, Kemal Aygun, Serife Solmaz, Asli Subasioglu, Saliha Aksun, Bahriye Payzin

**Affiliations:** 1 Department of Hematology, Izmir Katip Celebi University Ataturk Research and Training Hospital, 35730 Izmir, Turkey; tugbacetintepe@gmail.com (T.C.); alevgarip@yahoo.com (A.G.A.); drkml_aygun@hotmail.com (K.A.); solmazserife@yahoo.com (S.S.); bahriyepayzin@gmail.com (B.P.); 2 Department of Medical Biology, Faculty of Medicine, Ege University, 35100 Izmir, Turkey; royagasimli@gmail.com; 3 Department of Medical Genetics, Izmir Katip Celebi University Ataturk Research and Training Hospital, 35730 Izmir, Turkey; aslisubasioglu@gmail.com; 4 Department of Medical Biochemistry, Izmir Katip Celebi University Ataturk Research and Training Hospital, 35730 Izmir, Turkey; salihaaksun@yahoo.com

**Keywords:** MTHFR A1298C, MTHFR C677T, acute lymphoblastic leukemia, non-Hodgkin lymphoma

## Abstract

**Background/Objectives:** Methylenetetrahydrofolate reductase (MTHFR) gene polymorphisms may influence folate metabolism and DNA synthesis, potentially affecting disease characteristics and clinical outcomes in hematologic malignancies. This study investigated the associations of MTHFR C677T and A1298C polymorphisms with clinical features and survival outcomes in adult patients with acute lymphoblastic leukemia (ALL) or non-Hodgkin lymphoma (NHL). **Methods:** A total of 92 adult patients with ALL or NHL treated with standard chemotherapy were retrospectively analyzed. MTHFR C677T and A1298C genotypes were determined using real-time polymerase chain reaction. Associations between genotypes and baseline clinical features, treatment response, toxicity, overall survival (OS), and progression-free survival (PFS) were examined. **Results:** The C677T heterozygous genotype was significantly associated with the presence of B symptoms (*p* = 0.027). No significant differences were observed across genotypes with respect to other baseline clinical features, treatment response, or treatment-related toxicity. In the overall cohort (ALL + NHL), OS and PFS did not differ significantly by C677T or A1298C genotypes. However, in the NHL cohort, carriers of the C677T variant demonstrated significantly shorter PFS (*p* = 0.048) and a non-significant trend toward lower OS. This association was also observed in the DLBCL subgroup for PFS (*p* = 0.043), with a similar non-significant trend observed for OS. **Conclusions:** Although MTHFR genotyping appears to have limited value for broad clinical stratification, the observed association between the C677T polymorphism and PFS in NHL—particularly in the DLBCL subgroup—suggests a potential subtype-specific relevance that warrants further validation in larger, disease-specific cohorts.

## 1. Introduction

Methylenetetrahydrofolate reductase (MTHFR) gene polymorphisms may increase susceptibility to malignancies by disrupting DNA synthesis and repair through alterations in folate metabolism [1]. Since chemotherapeutic agents commonly act through inhibition of DNA synthesis, variations in folate pathways may influence treatment response, drug levels, and toxicity [2,3,4]. Therefore, MTHFR polymorphisms could carry clinical significance in hematologic malignancies, both through their role in tumor biology and in modulating chemotherapy metabolism [3,4].

MTHFR is located on chromosome 1p36.3 and encodes an enzyme essential for folate metabolism and DNA methylation [1,5]. The most common functional variants, C677T and A1298C, reduce enzyme activity to varying degrees [6,7] and have been associated with altered cancer risk in several solid tumors [8,9]. In hematologic malignancies, particularly acute lymphoblastic leukemia (ALL) and non-Hodgkin lymphoma (NHL), these polymorphisms have been investigated from multiple perspectives, including disease susceptibility, treatment-related toxicity, and survival outcomes. Most available studies have primarily focused on disease susceptibility; consequently, the direction and magnitude of reported associations have been inconsistent, with studies variably suggesting protective, adverse, or neutral effects, largely influenced by ethnic background and disease heterogeneity [10,11,12].

In recent years, MTHFR polymorphisms have been studied not only in relation to disease susceptibility but also for their possible associations with treatment-related toxicity and survival outcomes, with the majority of such studies conducted in pediatric populations [13,14,15,16]. This interest is particularly relevant for methotrexate (MTX), a key antifolate agent used in the treatment of ALL and selected NHL subtypes. Methotrexate inhibits dihydrofolate reductase, thereby interfering with folate-dependent DNA synthesis, while MTHFR plays a central role in intracellular folate homeostasis. Variations in MTHFR activity may therefore influence methotrexate pharmacokinetics, toxicity profiles, and therapeutic efficacy, as suggested by prior pharmacogenetic studies [2,3,4,14,15,16]. This biological interaction provides the rationale for evaluating methotrexate-related toxicity and treatment outcomes in relation to MTHFR polymorphisms in the present study. Nevertheless, outcome-oriented data in NHL—especially in adult patients—remain scarce, with available studies reporting heterogeneous results ranging from neutral associations to adverse effects on treatment response or survival.

Subtype-specific outcome data remain limited despite the marked biological and clinical heterogeneity of lymphoid malignancies. Most available studies evaluating MTHFR polymorphisms have analyzed heterogeneous patient populations without adequately accounting for differences between indolent and aggressive NHL subtypes, potentially obscuring subtype-specific associations with treatment response or survival. As a result, the clinical relevance of MTHFR variants in distinct lymphoma subgroups has not been clearly defined in adult patients.

Given this background, our study aimed to evaluate the clinical relevance of MTHFR C677T and A1298C polymorphisms in adult patients diagnosed with ALL or NHL. Specifically, we assessed their associations with baseline clinical and laboratory characteristics, treatment-related toxicity, including methotrexate-based analyses in a subset of patients, and survival outcomes. In addition to exploring their relationship with laboratory and clinical features, we examined their associations with progression-free survival (PFS) and overall survival (OS) within disease subtypes. Given the heterogeneity of lymphoid malignancies, particular attention was paid to more homogeneous lymphoma subgroups (e.g., diffuse large B-cell lymphoma and follicular lymphoma) in survival analyses.

## 2. Materials and Methods

### 2.1. Sample Collection and Genotyping

Patients aged ≥ 18 years who were diagnosed with ALL or NHL in our Hematology clinic between November 2021 and September 2024 and planned to receive first-line standard chemotherapy were included in the study. Patients with concomitant malignancies or additional hematologic disorders other than ALL or NHL were excluded.

Prior to initiating first-line chemotherapy, peripheral blood samples (2 mL) were collected in EDTA tubes. Genomic DNA was extracted using the EZ1 Advanced XL instrument (Qiagen, Hilden, Germany) with the EZ1&2 DNA Blood Kit (Cat. No: 951034; Qiagen, Hilden, Germany), according to the manufacturer’s instructions. Genotyping of MTHFR C677T and A1298C polymorphisms was performed by real-time polymerase chain reaction (qPCR) using the MTHFR (C677T) Real Time Kit (Cat. No: AA901) and MTHFR (A1298C) Real Time Kit (Cat. No: AA902) (Nuclear Laser Medicine S.r.l., Settala (MI), Italy). Amplification reactions were carried out on a Rotor-Gene Q instrument (Qiagen, Hilden, Germany) using the BA087 amplification enzyme (Nuclear Laser Medicine S.r.l., Settala (MI), Italy), according to the manufacturer’s protocol. Melting curve analysis and genotype determination were performed using Rotor-Gene Q Series Software (version 2.1.0.9, Qiagen, Hilden, Germany). For the A1298C variant, genotypes were classified as AA (wild type), AC (heterozygous), and CC (homozygous variant), whereas for the C677T variant, CC denoted the wild type, CT the heterozygous, and TT the homozygous variant genotype.

### 2.2. Clinical and Laboratory Data

Demographic and clinical information—including age, sex, diagnosis, chemotherapy regimen, number of treatment cycles, and thrombophilia history—was recorded. Laboratory parameters such as lymphocyte count, hemoglobin, platelet count, lactate dehydrogenase (LDH), erythrocyte sedimentation rate (ESR), vitamin B12, and folate levels were also documented. Plasma methotrexate (MTX) concentrations were monitored by routine assays performed in an external reference laboratory. Leucovorin rescue was initiated 12 h after MTX infusion and continued until MTX levels decreased below 0.1 µmol/L. Delayed MTX elimination was defined as plasma levels >1 µmol/L at 48 h or >0.2 µmol/L at 72 h post-infusion.

Adverse events, including grades 3–4 hematologic toxicities (anemia, neutropenia, lymphopenia, thrombocytopenia), mucositis, diarrhea, nephrotoxicity, hepatotoxicity, and other treatment-related toxicities, were monitored during the treatment course. Toxicities were graded according to the National Cancer Institute Common Terminology Criteria for Adverse Events (CTCAE, version 3.0), and for each patient the highest grade observed was used in the analysis. Treatment responses were categorized as complete response (CR), partial response (PR), or progressive disease (PD). Survival outcomes were evaluated as PFS and OS.

### 2.3. Statistical Analysis

All statistical analyses were performed using IBM SPSS Statistics for Windows, Version 25.0 (IBM Corp., Armonk, NY, USA). Descriptive statistics were presented as numbers and percentages for categorical variables and as mean ± standard deviation and median (min–max) for continuous variables. The Kolmogorov–Smirnov test was used to assess normality. As the assumption of normality was not met, the Kruskal–Wallis test was used for comparisons among three or more groups, with the Bonferroni correction applied for post hoc analysis. Pearson’s chi-square test or Fisher’s exact test was used for comparisons of categorical variables, as appropriate. Survival analyses for OS and PFS were performed using the Kaplan–Meier method and compared using the log-rank test. A *p*-value < 0.05 was considered statistically significant.

## 3. Results

### 3.1. Patient Characteristics

A total of 96 patients were enrolled in the study; however, 92 patients with complete diagnostic and treatment data were included in the final analysis. Of these, 64.1% (n = 59) were male and 35.9% (n = 33) were female, with a median age of 66 years (range: 21–93). Diagnoses included ALL in 9.8% (n = 9) and NHL in 90.2% (n = 83). Among the ALL patients, 77.8% (n = 7) had B-ALL and 22.2% (n = 2) had T-ALL. According to risk stratification, 33.3% (n = 3) of ALL patients were classified as standard risk and 66.7% (n = 6) as high risk. Among NHL subtypes, the distribution was as follows: diffuse large B-cell lymphoma (DLBCL) 44.6% (n = 37), follicular lymphoma (FL) 25.3% (n = 21), marginal zone lymphoma (MZL) 10.8% (n = 9), mantle cell lymphoma (MCL) 1.2% (n = 1), MALT lymphoma 1.2% (n = 1), CLL/SLL 2.4% (n = 2), Burkitt lymphoma 1.2% (n = 1), T-cell/histiocyte-rich large B-cell lymphoma (THRLBCL) 2.4% (n = 2), peripheral T-cell lymphoma NOS (PTCL-NOS) 2.4% (n = 2), anaplastic large cell lymphoma (ALCL) 4.8% (n = 4), cutaneous T-cell lymphoma (CTCL) 1.2% (n = 1), and NK/T-cell lymphoma 2.4% (n = 2). Risk stratification among NHL patients showed 25.3% as low risk, 33.7% low-intermediate risk, 19.3% intermediate–high risk, and 21.7% high risk. Patient characteristics by MTHFR A1298C and C677T genotype groups are summarized in Table 1 and Table 2. Genotype distributions are described in detail in the following section (Clinical and Laboratory Findings).

### 3.2. Clinical and Laboratory Findings

At diagnosis, B symptoms were present in 25% (n = 23) of patients. Bulky disease was observed in 18.5% (n = 17), bone marrow involvement in 25% (n = 23), central nervous system (CNS) involvement in 4.3% (n = 4), and splenomegaly in 23.9% (n = 22). A personal or family history of thrombophilia was noted in 4.3% (n = 4).

Baseline laboratory parameters for the overall cohort included median lymphocyte count of 1510/µL (range: 270–214,000), hemoglobin 12.2 g/dL (range: 4.6–15.7), platelet count 264 × 10^9^/L (range: 13–740), LDH 209 U/L (range: 96–2354), ESR 39 mm/h (range: 1–125), vitamin B12 214 pg/mL (range: 82–1163), and folic acid 6.7 ng/mL (range: 2.8–24.0). Laboratory parameters stratified according to MTHFR genotype are detailed in Table 1 (A1298C) and Table 2 (C677T). Subtype-specific baseline laboratory parameters for the major NHL subgroups (DLBCL and FL) are additionally provided in Supplementary Table S1.

Genotypic distribution analysis demonstrated the following frequencies: for MTHFR A1298C, AA (wild type) was observed in 41.3% (n = 38), AC (heterozygous) in 44.6% (n = 41), and CC (homozygous variant) in 14.1% (n = 13); for MTHFR C677T, CC (wild type) accounted for 51.1% (n = 47), CT (heterozygous) for 41.3% (n = 38), and TT (homozygous variant) for 7.6% (n = 7). Comparative analyses showed that, among all clinical and laboratory parameters, only the presence of B symptoms differed significantly across C677T genotype groups (χ^2^ = 7.23, *p* = 0.026).

B symptoms were more frequently observed in patients carrying the heterozygous CT genotype compared with the other C677T genotype groups, whereas no other significant associations were detected (*p* > 0.05). To further evaluate this finding, post hoc pairwise comparisons were performed. These analyses demonstrated that the statistical significance was mainly driven by the difference between heterozygous (CT) and wild-type (CC) genotypes (χ^2^ = 6.61, *p* = 0.010). No significant differences were detected between TT and CT genotypes (*p* = 0.200) or between TT and CC genotypes (*p* = 0.960). After Bonferroni correction for multiple comparisons, only the CT vs. CC comparison remained statistically significant (adjusted *p* = 0.030), whereas the other comparisons remained non-significant. Overall, these findings do not support a consistent allele–dose pattern. Detailed post hoc pairwise comparisons with Bonferroni-adjusted *p*-values are provided in Supplementary Table S2.

### 3.3. Subtype Distribution by Genotype

The distribution of NHL subtypes (DLBCL, FL, and others) was also evaluated by MTHFR mutation status. No significant associations were found between genotype and subtype distribution for either A1298C or C677T (*p* > 0.05). Detailed results are presented in Table 3.

### 3.4. Chemotherapy, Treatment Response, and Toxicity

To assess the clinical implications of MTHFR polymorphisms in therapeutic management, chemotherapy protocols, response outcomes, and adverse effects were examined. Among the 90 patients who received chemotherapy, 15.6% (n = 14) were treated with methotrexate (MTX)-containing regimens, whereas 84.4% (n = 76) received MTX-free protocols. The administered chemotherapy regimens were as follows: R-CHOP in 64.4% (n = 58), R-CVP in 7.8% (n = 7), R-CEOP in 1.1% (n = 1), R-bendamustine in 5.6% (n = 5), CHOP in 4.4% (n = 4), CHOEP in 2.2% (n = 2), Hyper-CVAD in 6.7% (n = 6), BFM in 3.3% (n = 3), high-dose MTX (HDMTX) in 2.2% (n = 2), and SMILE (dexamethasone, methotrexate, ifosfamide, L-asparaginase, and etoposide) in 2.2% (n = 2). Additionally, one patient with follicular lymphoma was managed with a watch-and-wait approach, and one patient declined chemotherapy because of advanced age. The median number of treatment cycles was 6 (range: 1–9). Grades 3–4 hematologic toxicities were common, including anemia (29.3%), neutropenia (19.5%), lymphopenia (37.7%), and thrombocytopenia (13.2%). Grades 1–2 mucositis was seen in 20.5% of patients; no cases of grades 3–4 mucositis occurred. No delays in MTX elimination were observed in patients who received MTX. Clinically significant diarrhea occurred in 5.6% (n = 5) of patients. No grades 3–4 hepatotoxicity or nephrotoxicity was observed. Treatment response was evaluated in 89 patients who received chemotherapy; one additional patient could not be evaluated because of early death during the initial cycles of treatment. Complete response (CR) was observed in 70.8% (n = 63), partial response (PR) in 9.0% (n = 8), and progressive disease (PD) in 20.2% (n = 18). No significant differences in toxicity or treatment response were observed across MTHFR genotypes (*p* > 0.05). Treatment response rates and toxicity outcomes according to MTHFR A1298C and C677T genotypes are also summarized in Table 1 and Table 2, respectively.

### 3.5. Survival Analysis

The median follow-up duration was 21.2 months (range: 1.1–48.4). During follow-up, relapse or progression occurred in 28.6% (n = 26) of patients. At the end of the study, 71.7% (n = 66) were alive, while 28.2% (n = 26) had died. Not all deaths were preceded by documented relapse or progression, as some patients died due to treatment-related complications such as infection during chemotherapy. Survival outcomes were analyzed, and median OS and PFS were not reached for the overall cohort comprising patients with ALL and NHL. In the overall cohort, no statistically significant differences in OS or PFS were observed according to MTHFR A1298C or C677T genotype groups (*p* > 0.05).

Given the predominance of NHL cases in the study population, subsequent survival analyses focused on the NHL cohort (Table 4). In this cohort, a significantly shorter PFS was observed in patients carrying the C677T mutation (CT + TT) compared with the wild-type group (2-year PFS 60.8% vs. 79.4%, *p* = 0.048). Although the difference in OS did not reach statistical significance, OS rates were numerically lower in patients with the C677T mutation than in wild-type patients (2-year OS 63.9% vs. 81.3%). No significant survival differences were observed according to the MTHFR A1298C genotype.

Subgroup analyses across NHL subtypes (DLBCL, FL, and other NHL) are presented in Table 5. In the DLBCL subgroup, a significantly lower 2-year PFS rate was observed in patients carrying the C677T mutation compared with the wild-type group (42.0% vs. 70.6%, *p* = 0.043). Although the difference in OS did not reach statistical significance (*p* = 0.224), patients with the C677T mutation also showed numerically lower 2-year OS rates than wild-type patients (55.7% vs. 83.3%). No statistically significant differences in OS or PFS were observed in FL or other NHL subtypes according to MTHFR genotype.

## 4. Discussion

Our findings contribute to the ongoing debate regarding the clinical relevance of MTHFR polymorphisms in hematologic malignancies. In the literature, studies investigating the relationship between MTHFR polymorphisms and hematological malignancies such as NHL and ALL have predominantly focused on the association between these genetic variants and disease susceptibility. Numerous publications have reported either a positive or null association between MTHFR C677T and A1298C polymorphisms and the risk of developing ALL or NHL in both pediatric and adult populations across different ethnic groups [17,18].

These inconsistencies are illustrated by several pooled analyses and population-based studies. For instance, a large meta-analysis including over 7800 ALL cases demonstrated a significant association between the MTHFR C677T T allele and increased susceptibility to ALL, particularly among Asian and pediatric populations [19]. A meta-analysis based on 28 case–control studies also confirmed this association, reporting a higher risk of ALL in Asian cohorts [20]. In contrast, other pooled analyses suggested a potential protective role of the T allele, especially in children [21,22]. Regarding NHL, a Japanese case–control study reported a reduced overall risk of malignant lymphoma among carriers of the MTHFR 677T or 1298C alleles, with histology-stratified analyses suggesting broadly similar patterns, although subtype-specific exceptions were noted [23]. However, evidence from ethnically diverse studies has yielded inconsistent results regarding the association between C677T and NHL risk, with several reports highlighting population-specific effects [11,18].

In our cohort, the distribution of MTHFR C677T and A1298C polymorphisms was further analyzed across different NHL subtypes. As presented in Table 3, no statistically significant differences were observed in the frequencies of these polymorphisms (*p* > 0.05). Due to the limited number of patients with ALL, genotype-based subgroup analysis was not performed for this disease group, and ALL cases were therefore not included in the comparative table. Moreover, the relatively small sample size within individual NHL subtypes, along with the absence of a matched healthy control group, should be considered when interpreting subtype-specific genotype distributions.

Notably, among all evaluated clinical and laboratory parameters, the presence of B symptoms differed significantly across C677T genotype groups (*p* = 0.026), with a higher frequency observed in patients carrying the heterozygous genotype. While the underlying mechanisms remain unclear, this association may reflect genotype-related differences in disease biology or host–tumor interactions. To our knowledge, data directly linking MTHFR genotype status to B symptom prevalence in lymphoid malignancies are scarce, highlighting the need for further studies to clarify the clinical and biological relevance of this observation.

In this context, detailed post hoc analyses indicated that this association was primarily driven by the heterozygous CT genotype, while no meaningful differences were observed between TT and CC genotypes. Moreover, an expected allele–dose relationship was not evident. If a true genotype-driven biological effect were present, a graded pattern across genotypes (e.g., TT > CT > CC or vice versa) would be anticipated. The absence of such a pattern, together with the limited sample size and uneven genotype distribution, suggests that this finding should be interpreted with caution. Accordingly, this association is best regarded as exploratory and hypothesis-generating rather than as definitive evidence of a true biological effect.

In contrast to the numerous studies evaluating MTHFR polymorphisms in the context of disease susceptibility, fewer investigations have addressed their potential impact on treatment-related toxicity and survival outcomes, particularly in adult populations. Some pediatric cohort studies have suggested an association between folate-pathway polymorphisms and HD-MTX-related toxicity [24,25], whereas large multicenter childhood analyses found no significant association [26]. Additionally, smaller pediatric case series have reported links between MTHFR variants and therapy-related toxicity [27].

In our study, only a small subset of patients (15.6%)—primarily those with acute lymphoblastic leukemia or central nervous system involvement—received high-dose MTX-containing treatment protocols. Among these, no cases of delayed MTX elimination or grades 3–4 mucositis or gastrointestinal toxicity were observed. The low number of patients exposed to MTX and the absence of relevant toxicities may have limited our ability to assess potential associations between MTHFR polymorphisms and MTX-related adverse events. Additionally, grades 3–4 hematologic toxicities (including anemia, neutropenia, and lymphopenia) were observed across the cohort, but no significant differences were noted between MTHFR genotype groups. These findings are consistent with previous adult series [14,28], which also reported no significant associations between MTHFR polymorphisms and MTX-related toxicity in patients with ALL or other hematologic malignancies.

Given the limited number of ALL cases and the predominance of NHL in our study population, survival analyses were primarily interpreted within the NHL cohort. Within this context, the C677T variant was the only polymorphism showing a statistically significant association with progression-free survival, accompanied by a non-significant trend toward lower overall survival. The observation of a similar pattern within the DLBCL subgroup suggests that this association may vary according to disease subtype rather than being uniform across all hematologic malignancies. In contrast, the A1298C variant did not appear to influence survival outcomes in our cohort.

The heterogeneous results reported in the literature are in line with this interpretation. Several adult series have described neutral findings [14], and Seidemann et al. [26] similarly reported no significant associations between MTHFR variants and survival outcomes in pediatric non-Hodgkin lymphoma. However, other studies have reported associations between the 677T allele and less favorable survival outcomes in selected clinical settings. For instance, an adult NHL cohort reported a lower probability of 5-year event-free survival among 677T-allele carriers, with borderline statistical significance, particularly in specific treatment contexts [28], while a prospective study in pediatric ALL patients found higher relapse rates and shorter relapse-free survival in individuals with the TT genotype, especially within high-risk groups [13]. Conversely, a large Chinese pediatric NHL cohort reported a more favorable event-free survival among T-allele carriers in certain subgroups [29]. In addition, a case–control study reported an association between the MTHFR 677C>T polymorphism and unfavorable outcomes in hematologic malignancies, including chronic myeloid leukemia [30]. Taken together, these data suggest that the clinical relevance of MTHFR C677T may be context-dependent, influenced by disease type, subtype, and treatment setting. Our findings in adult NHL, particularly in DLBCL, are consistent with this emerging pattern.

The differential impact observed between the two polymorphisms may be attributable to their respective biochemical consequences. The C677T variant leads to a substantial reduction in MTHFR enzymatic activity, which may impair folate metabolism, DNA methylation, and DNA repair processes that could potentially influence both disease progression and treatment efficacy. In contrast, the A1298C variant causes a milder reduction in enzyme function and may not significantly disrupt folate-related pathways. In our cohort, no meaningful associations were observed between the A1298C polymorphism and survival or treatment response, further supporting this functional distinction.

No significant differences in OS or PFS were observed according to either polymorphism among patients with FL or other NHL subtypes. The relatively indolent course of low-grade lymphomas, such as follicular lymphoma, may mask subtle genetic effects due to generally favorable outcomes and limited event rates.

Taken together, the findings of the present study indicate differential clinical associations between the two investigated MTHFR polymorphisms. While the A1298C variant showed no meaningful association with baseline clinical features, treatment response, treatment-related toxicity, or survival outcomes, this consistently negative result represents an important finding in itself. In contrast, the C677T polymorphism was the only variant showing a statistically significant association with progression-free survival in the NHL cohort. This association was primarily driven by patients with diffuse large B-cell lymphoma, whereas no statistically significant effect on overall survival was observed, despite a numerically inferior trend. Importantly, neither polymorphism demonstrated a significant relationship with treatment response or treatment-related toxicity. These findings underscore the subtype-specific and context-dependent nature of the observed associations.

In conclusion, while MTHFR polymorphisms do not appear to be uniformly associated with survival or treatment-related toxicity across adult ALL and NHL populations, our results suggest that the C677T variant may be associated with survival differences in specific clinical contexts, particularly in aggressive lymphoma subtypes such as DLBCL. The growing body of evidence linking this polymorphism to inferior outcomes in selected clinical settings is consistent with a potential role in disease progression and treatment response. These observations highlight the importance of further prospective and subtype-specific investigations to clarify the clinical relevance of MTHFR variants, rather than supporting their use for risk stratification in current practice.

## 5. Limitations

The relatively small number of patients with ALL and those receiving high-dose methotrexate limited the assessment of treatment-related toxicity in these subgroups. In addition, the modest sample size within individual NHL subtypes reduced the statistical power for subtype-specific analyses. Despite these limitations, the inclusion of a broad NHL cohort adds to the available evidence regarding the clinical relevance of MTHFR polymorphisms in adult lymphoid malignancies.

## Figures and Tables

**Table 1:** Comparison of Clinical and Laboratory Parameters According to MTHFR A1298C Genotype.

	A1298C			
Variables	Homozygous (CC) (n = 13)	Heterozygous (AC) (n = 41)	Wild Type (AA) (n = 38)	*p*-Value
Age, median (min–max)	60 (33–81)	66 (22–93)	66 (21–84)	0.376 ^c^
Sex, n (%)				
Female	5 (38.5)	16 (39)	12 (31.6)	0.771 ^a^
Male	8 (61.5)	25 (61)	26 (68.4)	
Diagnose				
ALL	1 (7.7)	4 (9.8)	4 (10.5)	-
NHL	12 (92.3)	37 (90.2)	34 (89.5)	
ALL subtype				
B-ALL	1 (100)	3 (75)	3 (75)	-
T-ALL	0 (0)	1 (25)	1 (25)	
ALL risk group				
Standart risk	0 (0)	2 (50)	2 (40)	-
High risk	1 (100)	2 (50)	3 (60)	
NHL risk group				
Low	3 (25)	7 (18.9)	11 (32.4)	0.679 ^b^
Low-Intermediate	5 (41.7)	12 (32.4)	11 (32.4)	
Intermediate-High	3 (25)	7 (18.9)	6 (17.6)	
High	1 (8.3)	11 (29.7)	6 (33.3)	
B symptoms				
No	11 (84.6)	30 (73.2)	28 (73.7)	0.688 ^a^
Yes	2 (15.4)	11 (26.8)	10 (26.3)	
Bulky disease				
No	10 (76.9)	35 (85.4)	30 (78.9)	0.687 ^a^
Yes	3 (23.1)	6 (14.6)	8 (21.1)	
Bone Marrow Involvement				
No	10 (76.9)	31 (75.6)	28 (73.7)	0.966 ^a^
Yes	3 (23.1)	10 (24.4)	10 (26.3)	
CNS Involvement				
No	13 (100)	39 (95.1)	36 (94.7)	0.721 ^b^
Yes	0 (0)	2 (4.9)	2 (5.3)	
History of Thrombophilia				
No	13 (100)	39 (95.1)	36 (94.7)	0.720 ^b^
Yes	0 (0)	2 (4.9)	2 (5.3)	
Splenomegaly				
No	8 (61.5)	35 (85.4)	27 (71.1)	0.137 ^a^
Yes	5 (38.5)	6 (14.6)	11 (28.9)	
Laboratory (median (min–max)				
Lymphocyte (10^9^/L)	1.43 (0.65–4.51)	1.34 (0.27–105)	1.55 (0.44–66.46)	0.687 ^c^
Hemoglobin (gr/dL)	11.9 (9.7–14.4)	12.2 (7.9–15.1)	12.4 (4.6–14.7)	0.122 ^c^
Platelets (10^9^/L)	342 (72–740)	286.5 (102–739)	243 (24–426)	0.111 ^c^
LDH (IU/L)	196 (136–374)	214 (96–2354)	225 (128–445)	0.833 ^c^
ESR (mm/h)	57 (21–114)	34 (1–125)	43 (7–104)	0.153 ^c^
Vitamin B12 (pg/mL)	176 (115–384)	220.5 (82–587)	219 (109–1163)	0.205 ^c^
Folic acid (ng/mL)	6.1 (3.4–16.1)	6.8 (2.8–24)	6.0 (3.5–23)	0.407 ^c^
Grades 3–4 Hematologic AE				
No	4 (30.8)	7 (17.1)	7 (18.4)	0.541 ^a^
Yes	9 (69.2)	34 (82.9)	31 (81.6)	
Mucositis				
No	11 (84.6)	33 (80.5)	30 (78.9)	0.906 ^a^
Grades 1–2	2 (15.4)	8 (19.5)	8 (21.1)	
Response to Treatment				
CR	8 (66.7)	27 (67.5)	28 (75.7)	0.759 ^b^
PR	2 (16.7)	4 (10)	2 (5.4)	
PD	2 (16.7)	9 (22.5)	7 (18.9)	

Statistical Tests Used: ^a^ Pearson Chi-Square test, ^b^ Fisher’s Exact test, ^c^ Kruskal–Wallis test; *p* < 0.05 = significant. Due to the limited number of ALL cases (n = 9), analyses in this subgroup were descriptive.

**Table 2:** Comparison of Clinical and Laboratory Parameters According to MTHFR C677T Genotype.

	C677T			
Variables	Homozygous (TT) (n = 7)	Heterozygous (CT) (n = 38)	Wild Type (CC) (n = 47)	*p*-Value
Age, median (min–max)	67 (34–77)	66 (22–87)	66 (21–93)	0.891 ^c^
Sex, n (%)				
Female	2 (28.6)	14 (36.8)	17 (36.2)	0.866 ^b^
Male	5 (71.4)	24 (63.2)	30 (63.8)	
Diagnose				
ALL	1 (14.3)	3 (7.9)	5 (10.6)	-
NHL	6 (85.7)	35 (92.1)	42 (89.4)	
ALL subtype				
B-ALL	1 (100)	2 (66.7)	4 (80)	-
T-ALL	0 (0)	1 (33.3)	1 (20)	
ALL risk group				
Standart risk	0 (0)	2 (66.7)	2 (33.3)	-
High risk	1 (100)	1 (33.3)	4 (66.7)	
NHL risk group				
Low	2 (33.3)	6 (17.1)	13 (31)	0.671 ^b^
Low-Intermediate	3 (50)	13 (37.1)	12 (28.6)	
Intermediate-High	0 (0)	7 (20)	9 (21.4)	
High	1 (16.7)	9 (25.7)	8 (44.4)	
B symptoms				
No	6 (85.7)	23 (60.5)	40 (85.1)	0.026 ^a^
Yes	1 (14.3)	15 (39.5)	7 (14.9)	
Bulky disease				
No	5 (71.4)	34 (89.5)	36 (76.6)	0.243 ^a^
Yes	2 (28.6)	4 (10.5)	11 (23.4)	
Bone Marrow Involvement				
No	5 (71.4)	27 (71.1)	37 (78.7)	0.701 ^a^
Yes	2 (28.6)	11 (28.9)	10 (21.3)	
CNS Involvement				
No	6 (85.7)	37 (97.4)	45 (95.7)	0.366 ^b^
Yes	1 (14.3)	1 (2.6)	2 (4.3)	
History of Thrombophilia				
No	6 (85.7)	36 (94.7)	46 (97.9)	0.263 ^b^
Yes	1 (14.3)	2 (5.3)	1 (2.1)	
Splenomegaly				
No	4 (57.1)	29 (76.3)	37 (78.7)	0.458 ^a^
Yes	3 (42.9)	9 (23.7)	10 (21.3)	
Laboratory, median (min–max)				
Lymphocyte (10^9^/L)	1.95 (0.46–15.8)	1.42 (0.27–66.5)	1.42 (0.42–105)	0.836 ^c^
Hemoglobin (gr/dL)	12.4 (8.3–14.7)	12.1 (4.6–15.1)	12.4 (7.9–15.1)	0.590 ^c^
Platelets (10^9^/L)	218 (24–412)	285 (80–456)	288 (72–740)	0.321 ^c^
LDH (IU/L)	246 (148–412)	201 (141–997)	204.5 (96–2354)	0.829 ^c^
ESR (mm/h)	19 (12–104)	44 (7–125)	36 (1–114)	0.627 ^c^
Vitamin B12 (pg/mL)	197 (113–445)	209 (113–1163)	224 (82–773)	0.676 ^c^
Folic acid (ng/mL)	5,4 (3.5–10.5)	6.7 (3.3–24)	6.6 (2.8–23)	0.903 ^c^
Grades 3–4 Hematologic AE				
No	3 (42.9)	8 (21.1)	7 (14.9)	0.210 ^a^
Yes	4 (57.1)	30 (78.9)	40 (85.1)	
Mucositis				
No	5 (71.4)	32 (84.2)	37 (78.7)	0.673 ^a^
Grades 1–2	2 (28.6)	6 (15.8)	10 (21.3)	
Response to Treatment				
CR	4 (66.7)	24 (63.2)	35 (77.8)	0.350 ^b^
PR PD	1 (16.7) 1 (16.7)	3 (7.9) 11 (28.9)	4 (8.9) 6 (13.3)	

Statistical Tests Used: ^a^ Pearson Chi-Square test, ^b^ Fisher’s Exact test, ^c^ Kruskal–Wallis test; *p* < 0.05 = significant. Due to the limited number of ALL cases (n = 9), analyses in this subgroup were descriptive.

**Table 3:** Distribution of NHL Subtypes According to MTHFR A1298C and C677T Genotypes.

	A1298C			C677T		
Variables	Mutated (CC + AC) n = 49 (%)	Wild Type (AA) n = 34 (%)	*p*	Mutated (TT + CT) n = 41 (%)	Wild Type (CC) n = 42 (%)	*p*
NHL subtype						
DLBCL	24 (49.0)	13 (38.2)	0.588	19 (46.4)	18 (42.9)	0.810
FL	12 (24.5)	9 (26.5)		11 (26.8)	10 (23.8)	
Other NHL	13 (26.5)	12 (35.3)		11 (26.8)	14 (33.3)	

Data are presented as n (%). Pearson’s chi-square test was used for comparisons; *p* < 0.05 was considered statistically significant.

**Table 4:** Overall Survival (OS) and Progression-Free Survival (PFS) According to MTHFR A1298C and C677T Genotypes in the NHL Cohort.

Variables	2-Year OS %	Median OS (%95 CI)	*p* Value	2-Year PFS %	Median PFS (%95 CI)	*p* Value
Overall	72.9	- (-)		70.2	- (-)	
A1298C						
Homozygous (CC)	77.9	- (-)	0.786	65.5	- (-)	0.894
Heterozygous (AC)	69.7	- (-)		67.3	- (-)	
Wild Type (AA)	75.3	- (-)		74.4	- (-)	
Mutated (CC + AC)	71.3	- (-)	0.593	66.9	- (-)	0.636
Wild Type (AA)	75.3	- (-)		74.4	- (-)	
C677T						
Homozygosus (CC)	62.5	- (-)	0.451	26.7	22.3 (12.1–32.6)	0.127
Heterozygous (CT)	64.6	- (-)		65.6	- (-)	
Wild Type (CC)	81.3	- (-)		79.4	- (-)	
Mutated (TT + CT)	63.9	- (-)	0.215	60.8	- (-)	0.048
Wild Type (CC)	81.3	- (-)		79.4	- (-)	

Kaplan–Meier method with log-rank test. Statistical significance was defined as *p* < 0.05. Analyses were performed in the NHL cohort. Note: “- (-)” indicates that the median survival was not reached during the follow-up period; the median value and corresponding 95% confidence intervals could not be calculated.

**Table 5:** Overall Survival (OS) and Progression-Free Survival (PFS) According to MTHFR Polymorphisms Across NHL Subtypes.

OS	DLBCL			FL			Other NHL		
MTHFR Genotypes	2-year OS %	Median OS (%95 CI)	*p*	2-year OS %	Median OS (%95 CI)	*p*	2-year OS %	Median OS (%95 CI)	*p*
A1298C									
CC + AC (mutated)	73.7	- (-)	0.941	82.5	- (-)	0.721	66.7	- (-)	0.596
AA (wild type)	64.5	- (-)		83.3	- (-)		80.2	- (-)	
C677T									
TT + CT (mutated)	55.7	- (-)	0.224	85.7	- (-)	0.494	56.8	- (-)	0.227
CC (wild type)	83.3	- (-)		78.8	- (-)		83.3	- (-)	
PFS	DLBCL			FL			Other NHL		
	2-year PFS %	Median PFS (%95 CI)	*p*	2-year PFS %	Median PFS (%95 CI)	*p*	2-year PFS %	Median PFS (%95 CI)	*p*
A1298C									
CC + AC (mutated)	53.9	- (-)	0.920	82.5	- (-)	0.820	66.7	- (-)	0.488
AA (wild type)	57.0	- (-)		85.7	- (-)		83.3	- (-)	
C677T									
TT + CT (mutated)	42.0	22.36 (3.88–40.84)	0.043	88.9	- (-)	0.545	60.6	- (-)	0.200
CC (wild type)	70.6	- (-)		78.8	- (-)		84.6	- (-)	

Kaplan–Meier method and log-rank test. *p* < 0.05 was considered statistically significant. Analyses were performed across NHL subtypes. Note: “- (-)” indicates that the median survival was not reached during the follow-up period; the median value and corresponding 95% confidence intervals could not be calculated.

## Data Availability

The data that support the findings of this study are available from the corresponding author upon reasonable request.

## Supplementary Materials

The following supporting information can be downloaded at: https://www.mdpi.com/article/10.3390/jcm15051796/s1, Table S1: Subtype-specific baseline laboratory parameters according to MTHFR A1298C and C677T genotypes in major NHL subgroups (DLBCL and FL); Table S2: Post-hoc pairwise comparisons of MTHFR C677T genotypes for the presence of B symptoms with Bonferroni adjustment.
