# Supplementary material for: Clinical Significance of MTHFR C677T and A1298C Polymorphisms in Adult Patients with ALL and NHL
Source: J Clin Med. 2026 Feb 27;15(5):1796. doi: 10.3390/jcm15051796 (PMC12985645; doi:10.3390/jcm15051796)
Supplement: Supplementary file 1 [file jcm-15-01796-s001.zip › jcm-4134211-supplementary.pdf]

Supplementary Table S1.

Subtype-specific baseline laboratory parameters according to MTHFR A1298C and C677T genotypes in major NHL subgroups (DLBCL and FL).

		A1298C			
	**Variables**	**Homozygous (CC)**	**Heterozygous (AC)**	**Wild Type (AA)**	**p-value**
DLBCL	Laboratory. median (min-max				
	**Lymphocyte (10^9/l)	1430 (970.0-2230.0)	1200 (270-2440)	1115 (440-2990)	0.808[c]
	**Hemoglobin (gr/dl)	10.4 (9.7-12.8)	12.2 (8.5-15.1)	12.2 (4.6-14.7)	0.289[c]
	**Platelets (10^9/l)	357 (268-740)	334 (164-739)	240.5 (128-332)	**0.003[c]**
	**LDH (IU/l)	228 (136-374)	198 (124-997)	223 (146-445)	0.824[c]
	**ESR (mm/h)	104 (34-114)	47 (11-97)	44.5 (11-95)	**0.043[c]**
	**Vitamin B12 (pg/ml)	145 (115-384)	209 (82-587)	223 (113-598)	0.692[c]
	**Folic acid (ng/ml)	4.9 (3.4-11.4)	6.8 (3.3-23)	5.8 (3.5-10.5)	0.724[c]
FL	Laboratory. median (min-max				
	**Lymphocyte (10^9/l)	1325 (650-2000)	1565 (440-2710)	2790 (510-66460)	0.251[c]
	**Hemoglobin (gr/dl)	12.9 (12.1-13.8)	13.2 (7.9-14.6)	12.4 (9.2-13.6)	0.584[c]
	**Platelets (10^9/l)	192 (72-312)	241 (145-417)	168 (80-426)	0.697[c]
	**LDH (IU/l)	253 (196-311)	243 (182-2354)	225 (128-397)	0.669[c]
	**ESR (mm/h)	49.5 (21-78)	23.5 (19-113)	44 (17-75)	0.426[c]
	**Vitamin B12 (pg/ml)	168.5 (143-194)	278 (107-470)	252 (109-625)	0.512[c]
	**Folic acid (ng/ml)	11.5 (6.9-16.1)	6.5 (4.3-8.1)	5.4 (4.3-7.9)	**0.012[c]**
		**C677T**			
	**Variables**	**Homozygous (TT)**	**Heterozygous (CT)**	**Wild Type (CC)**	**p-value**
DLBCL	Laboratory. median (min-max				
	**Lymphocyte (10^9/l)	1545 (460–2990)	1200 (270–2610)	1360 (420–2440)	0.466[c]
	**Hemoglobin (gr/dl)	11.6 (8.3–14.7)	12.0 (4.6–15.1)	12.2 (8.5–15.1)	0.682[c]
	**Platelets (10^9/l)	229 (133–267)	332 (128–456)	301 (164–740)	0.161[c]
	**LDH (IU/l)	208.5 (148–412)	234 (146–997)	190 (124–472)	0.528[c]
	**ESR (mm/h)	16 (12–47)	49 (11–95)	54 (11–114)	0.116[c]
	**Vitamin B12 (pg/ml)	201.5 (113–219)	227 (156–598)	200 (82–437)	0.127[c]
	**Folic acid (ng/ml)	5.8 (3.5–10.5)	5.9 (3.3–23.0)	6.7 (3.4–12.2)	0.938[c]
FL	Laboratory. median (min-max				
	**Lymphocyte (10^9/l)	1880 (950–15810)	2490 (1400–66460)	1280 (440–2790)	0.376[c]
	**Hemoglobin (gr/dl)	13.4 (13.2–13.6)	12.3 (9.2–14.6)	12.4 (7.9–14.5)	0.704[c]
	**Platelets (10^9/l)	315 (218–412)	152 (80–417)	288 (72–426)	0.325[c]

**LDH (IU/l)	248 (225–271)	228.5 (141–397)	196 (128–2354)	0.728[c]
**ESR (mm/h)	40 (17–63)	38 (19–75)	26 (18–113)	0.907[c]
**Vitamin B12 (pg/ml)	306 (167–445)	247.5 (113–423)	194 (107–625)	0.943[c]
**Folic acid (ng/ml)	5.3 (5.2–5.4)	5.6 (4.3–8.1)	7.5 (4.3–16.1)	0.243[c]

Abbreviations: DLBCL, diffuse large B-cell lymphoma; FL, follicular lymphoma; LDH, lactate dehydrogenase; ESR, erythrocyte sedimentation rate.

Data are presented as median (minimum–maximum).

[c] p-values were calculated using the Kruskal–Wallis test.

Supplementary Table S2.

Post-hoc pairwise comparisons of MTHFR C677T genotypes for the presence of B symptoms with Bonferroni adjustment

Comparison	χ^2	p-value	Bonferroni-adjusted p
CT vs CC	6.61	p = 0.010	p = 0.030
TT vs CT	1.63	p = 0.200	p = 0.600
TT vs CC	0.0018	p = 0.960	p = 1.000

χ^2: chi-square statistic.
